# Supplementary material for: Training intensity and improvements in exercise capacity in elderly patients undergoing European cardiac rehabilitation – the EU-CaRE multicenter cohort study
Source: PLoS One. 2020 Nov 13;15(11):e0242503. doi: 10.1371/journal.pone.0242503 (PMC7665625; doi:10.1371/journal.pone.0242503)
Supplement: S1 Fig — (DOCX) [file pone.0242503.s001.docx]

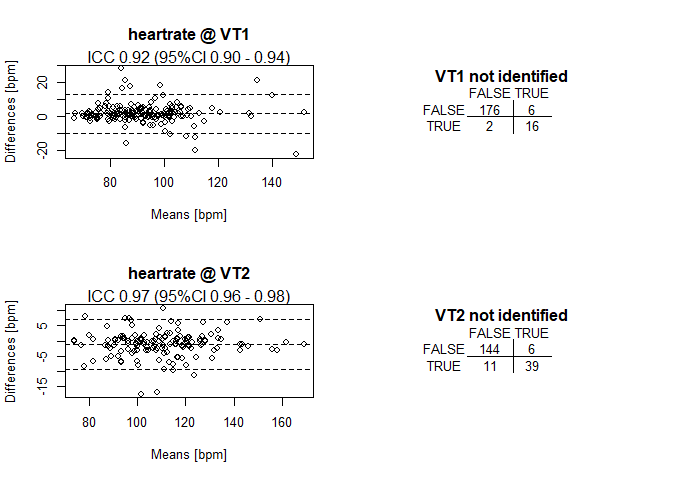


S1 Fig: Reproducibilty of ventilatory thresholds setting in a subset of 200 randomly chosen cardiopulmonary exercise tests. Shown are Bland-Altman plots and the ICC (single fixed raters) for the level of agreement between examiner 1 (TM) and examiner 2 (MW) on the left side. Cross-tables on the right depict the number of tests for which examiner 1 and/or examiner 2 could not identify a ventilatory threshold.

VT, ventilatory threshold; ICC, intraclass coefficient (single fixed raters); CI, confidence interval.
