# Supplementary material for: Training intensity and improvements in exercise capacity in elderly patients undergoing European cardiac rehabilitation – the EU-CaRE multicenter cohort study
Source: PLoS One. 2020 Nov 13;15(11):e0242503. doi: 10.1371/journal.pone.0242503 (PMC7665625; doi:10.1371/journal.pone.0242503)
Supplement: S1 Table — (DOCX) [file pone.0242503.s002.docx]

S1 Table. Linear mixed model on change in peak VO2 [ml/kg/min] with

centre as random factor:

---------------------------------------------------------------------

Intercept 2.06 [ 1.28; 2.84] *

Training above VT1 0.62 [ 0.24; 1.00] *

Total training volume per CR [h] 0.06 [ 0.01; 0.12] *

Duration of cardiac rehabilitation [days] 0.00 [-0.00; 0.01]

baseline VO2 [ml/kg/min] -0.09 [-0.13; -0.05] *

Age [years] -0.08 [-0.11; -0.04] *

stable CAD (ref. CABG) -1.31 [-2.10; -0.52] *

PCI (ref. CABG) -1.50 [-1.95; -1.05] *

percutaneous HVR (ref. CABG) -2.02 [-3.67; -0.37] *

surgical HVR (ref. CABG) -0.13 [-0.90; 0.64]

sex 0.60 [ 0.10; 1.10] *

Betablocker treatment 0.08 [-0.38; 0.55]

---------------------------------------------------------------------

AIC 3852.50

BIC 3918.22

Log Likelihood -1912.25

Num. obs. 808

Num. groups: siteid 7

Var: siteid (Intercept) 0.14

Var: Residual 6.42

=====================================================================

* 0 outside the 95% confidence interval
