# Supplementary material for: Training intensity and improvements in exercise capacity in elderly patients undergoing European cardiac rehabilitation – the EU-CaRE multicenter cohort study
Source: PLoS One. 2020 Nov 13;15(11):e0242503. doi: 10.1371/journal.pone.0242503 (PMC7665625; doi:10.1371/journal.pone.0242503)
Supplement: Attachment — Submitted filename: Response to the Editor and Reviewers.docx [file pone.0242503.s003.docx]

Response to the Editor and Reviewers

We would like to thank the reviewers for the very valuable comments, which we have gladly addressed and hopefully implemented to the editor’s and reviewers’ expectations. Many comments were very valid and we feel that the presented changes have greatly improved the clarity of the paper. Please find our point-to-point responses in red below.

**Journal requirements**

1. Please ensure that your manuscript meets PLOS ONE's style requirements, including those for file naming. The PLOS ONE style templates can be found at

<https://journals.plos.org/plosone/s/file?id=wjVg/PLOSOne_formatting_sample_main_body.pdf> and

<https://journals.plos.org/plosone/s/file?id=ba62/PLOSOne_formatting_sample_title_authors_affiliations.pdf>

We have adapted the manuscript style according to the template.

2. Thank you for stating the following in the Competing Interest section:

"AWJVH reports grants from Medtronic, grants and personal fees from Astra Zeneca, outside the submitted work, UZ reports grants and personal fees from Astra Zeneca, grants and personal fees from Bayer, personal fees from Boehringer Ingelheim, grants and personal fees from BMS, personal fees from Daiichi Sankyo, personal fees from Eli Lilly, grants and personal fees from Novartis, grants and personal fees from MSD, personal fees from Trommsdorf, personal fees from Amgen, outside the submitted work
All other authors have no Conflict of Interest to declare."

We note that one or more of the authors are employed by a commercial company: Diagram B.V., Zwolle.

Diagram B.V. is a contract research organization, however, the present study was an observational study on effects of clinically indicated CR, so there was neither an intervention nor a treatment involving any commercial interests. Diagram received the salaries for L.P. and E.K. from the EC Horizon2020 grant.

2.1. Please provide an amended Funding Statement declaring this commercial affiliation, as well as a statement regarding the Role of Funders in your study. If the funding organization did not play a role in the study design, data collection and analysis, decision to publish, or preparation of the manuscript and only provided financial support in the form of authors' salaries and/or research materials, please review your statements relating to the author contributions, and ensure you have specifically and accurately indicated the role(s) that these authors had in your study. You can update author roles in the Author Contributions section of the online submission form.

We have amended the Funding Statement accordingly.

Please also include the following statement within your amended Funding Statement.

“The funder provided support in the form of salaries for authors [insert relevant initials], but did not have any additional role in the study design, data collection and analysis, decision to publish, or preparation of the manuscript. The specific roles of these authors are articulated in the ‘author contributions’ section.”

We have amended the Funding Statement accordingly.

If your commercial affiliation did play a role in your study, please state and explain this role within your updated Funding Statement.

None of the commercial affiliations or supports played any role in this study. This is now also stated in the Funding Section, which now reads:

“For the Swiss consortium partner (TM, PE, MW), funding was received by the Swiss State Secretariat for Education, Research and Innovation under contract number 15.0139. All other authors received funding by the European Union’s Horizon 2020 research and innovation programme under grant agreement No 634439. The funders provided support in the form of salaries for authors [TM, PE, LP, EK, WB, AvV], but had no additional role in study design, data collection and analysis, decision to publish, or preparation of the manuscript. None of the commercial partners who provided financial support to any of the coauthors had any role in the perception, conduction, analysis, interpretation nor dissemination of this study.”

2.2. Please also provide an updated Competing Interests Statement declaring this commercial affiliation along with any other relevant declarations relating to employment, consultancy, patents, products in development, or marketed products, etc.

Within your Competing Interests Statement, please confirm that this commercial affiliation does not alter your adherence to all PLOS ONE policies on sharing data and materials by including the following statement: "This does not alter our adherence to  PLOS ONE policies on sharing data and materials.” (as detailed online in our guide for authors [http://journals.plos.org/plosone/s/competing-interests](about:blank)) . If this adherence statement is not accurate and  there are restrictions on sharing of data and/or materials, please state these. Please note that we cannot proceed with consideration of your article until this information has been declared.

Please include both an updated Funding Statement and Competing Interests Statement in your cover letter. We will change the online submission form on your behalf.

Please know it is PLOS ONE policy for corresponding authors to declare, on behalf of all authors, all potential competing interests for the purposes of transparency. PLOS defines a competing interest as anything that interferes with, or could reasonably be perceived as interfering with, the full and objective presentation, peer review, editorial decision-making, or publication of research or non-research articles submitted to one of the journals. Competing interests can be financial or non-financial, professional, or personal. Competing interests can arise in relationship to an organization or another person. Please follow this link to our website for more details on competing interests: [http://journals.plos.org/plosone/s/competing-interests](about:blank)

3. We note that you have indicated that data from this study are available upon request. PLOS only allows data to be available upon request if there are legal or ethical restrictions on sharing data publicly. For information on unacceptable data access restrictions, please see <http://journals.plos.org/plosone/s/data-availability#loc-unacceptable-data-access-restrictions>.

In your revised cover letter, please address the following prompts:

a) If there are ethical or legal restrictions on sharing a de-identified data set, please explain them in detail (e.g., data contain potentially identifying or sensitive patient information) and who has imposed them (e.g., an ethics committee). Please also provide contact information for a data access committee, ethics committee, or other institutional body to which data requests may be sent.

b) If there are no restrictions, please upload the minimal anonymized data set necessary to replicate your study findings as either Supporting Information files or to a stable, public repository and provide us with the relevant URLs, DOIs, or accession numbers. Please see <http://www.bmj.com/content/340/bmj.c181.long> for guidelines on how to de-identify and prepare clinical data for publication. For a list of acceptable repositories, please see <http://journals.plos.org/plosone/s/data-availability#loc-recommended-repositories>.

“We have included the following Conflict of Interest and Data sharing statements in our cover letter:

AWJVH reports grants from Medtronic, grants and personal fees from Astra Zeneca, outside the submitted work, UZ reports grants and personal fees from Astra Zeneca, grants and personal fees from Bayer, per-sonal fees from Boehringer Ingelheim, grants and personal fees from BMS, personal fees from Daiichi Sankyo, personal fees from Eli Lilly, grants and personal fees from Novartis, grants and personal fees from MSD, personal fees from Trommsdorf, personal fees from Amgen, outside the submitted work. LP and EK work for Diagram B.V., a contract research organization. These commercial affiliations do not alter our adherence to all PLOS ONE policies on sharing data and materials. All other authors have no Conflict of Interest to declare.”

There are, however, restrictions to data sharing but these are based on the various legislative restrictions for data containing potentially identifying or sensitive patient information of the involved eight countries and the lack of informed consent to international data sharing. Requests for de-identified data may be sent to Prof. Arnoud Van’t Hoff, Department of Cardiology, Maastricht University Medical Center, and Cardiovas-cular Research Institute Maastricht (CARIM), the Netherlands

We will update your Data Availability statement on your behalf to reflect the information you provide.

4. Please include captions for your Supporting Information files at the end of your manuscript, and update any in-text citations to match accordingly. Please see our Supporting Information guidelines for more information: [http://journals.plos.org/plosone/s/supporting-information](about:blank).

**Reviewer #1:** The subject of this study is very relevant at this moment: I therefor thank the authors for performing this analysis: the percentage of (elderly) patients attending and completing CR remains too low worldwide.
The following items I think is missing/unanswered:
Was there a difference in results between pts LVEF above vs below 40% or between ACS vs stable AP vs valvereplacement pts?

Change in peak VO2:

Another manuscript *(“Predictors for one-year outcomes of cardiorespiratory fitness and cardiovascular risk factor control after cardiac rehabilitation in elderly patients: the EU-CaRE study»)* has been submitted to Annals of Physical and Rehabilitation Medicine. Within this manuscript, extensive analyses were performed to identify predictors for change in peak VO2 as well as secondary outcomes. Chronic heart failure or ACS were not significant predictors in the model for change in peak VO2 when adjusted for age, sex, as well as multiple other comorbidities and cardiovascular risk factors. However, patients after cardiac surgery showed greater improvements than patients who had only a minimal invasive procedure (PCI, TAVI) or no procedure (chronic CAD). The greater improvement in surgery patient has also been discussed in a previous publication: *Prescott et al. Cardiac rehabilitation of elderly patients in eight rehabilitation units in Western Europe: Outcome data from the EU-CaRE multi-centre observational study. Eur J Prev Cardiol. 2020 Nov;27(16):1716-1729. doi: 10.1177/2047487320903869. Epub 2020 Feb 26. PMID: 32102550.*

Effect of training intensity on change in peak VO2:

The present analysis on the effect of training intensity (above versus below the individual first ventilatory threshold) has been adjusted for index intervention (PCI, chronic CAD, CABG and heart valve replacement). Based on your comment, we included the interaction between index intervention * training intensity as well as LVEF * training intensity into the model. Both interaction terms were not significant (p = 0.340 and 0.390, respectively). Hence, we do not have evidence that the effect of the training intensity on change in peak VO2 is different in patients with reduced LVEF or affected by the diagnosis.

We suggest to leave the manuscript with regard to these points as it is, because 1) the effect of patient characteristics on change in peak VO2 is discussed in great detail within another manuscript, and 2) neither interaction of training intensity with index intervention nor LVEF on change in peak were significant and inclusion of these interaction terms would make the statistical analysis section more difficult to comprehend.

What was the percentage of patients who completed the whole CR program offered per center?

Another manuscript (“*Offering, participation and adherence to cardiac rehabilitation programmes in the elderly: a European comparison*”) focusing on the participation rate and adherence to the CR programs has recently been accepted by the European Journal of Preventive Medicine. The participation rate averaged across all centers was 68% and adherence to CR was 90.3%.

Why was the ventilator thresholds determined by 1 single investigator only?

The ventilatory thresholds were also determined by the clinicians of the respective centres as part of clinical routine. However, we felt that this would increase the risk of a systematic bias, e.g., some centers would achieve systematically higher thresholds than others. In order to apply the same criteria to all patients from all centres we chose one single investigator set all thresholds in a random and blinded fashion on blinded raw data images (after applying some filtering and smoothing). This should be viewed as a strength rather than a limitation. However, we agree with the reviewer that some quality check was necessary and have added a sub-analysis of 200 CPETs analysed by another experienced cardiologist (MW) to assess inter-rater reliability (Supplement Figure 1). With an Intraclass-Correlation-Coefficient of 0.92 for VT1 and 0.97 for VT2 the reliability was excellent.

Do you think there was a selection bias because only patients with monitored training sessions, and only those with good quality CPET (that allowed the determination of VT1) could be included in the multivariate models? Did this greatly influenced your results?

We believe that we have already included our judgement of selection bias. Please note the following paragraph in the limitation section:

*Additionally, results of the present study do not reflect the whole population of the EU-CaRE study as only patients with monitored training sessions, and only those with good quality CPET (that allowed the determination of VT1) could be included in the multivariate models. Baseline peak VO2 of the patients included in the model was 17.36 ml/kg/min, while the mean baseline peak VO2 of all EU-CaRE patients was 15.94 ml/kg/min.*

This indicates that there may indeed be a selection bias for our analysis. It seems that ventilatory thresholds are more difficult to detect in patients with low fitness. However, it is difficult to speculate on how this selection bias may influence our results. Namely, in the related study on predictors of change in peak VO2 with CR we found baseline peak VO2 to be negatively related to change in peak VO2 in non-surgery patients and unrelated to surgery patients (unpublished data). In our view, the study results reflect how difficult it often is to set valid ventilatory thresholds in clinical routine, especially in elderly patients with reduced physical function.

You advocate " self-paced-intensity", especially because this is an elderly population: I completely agree: do you know what the relation was between the achieved training intensities and improvement in Quality of Life in your study group?

Thank you for this interesting question. Self-reported QoL was assessed by the SF36 questionnaire resulting in a physical (PCS) and mental (MCS) component score. The MCS at baseline and change in MCS over time did not differ between patients exercising above or below the VT. However, patients who exercised above the VT1 had higher PCS score at baseline (p = 0.046) and tended to improve their PCS score more (p = 0.06). We believe that it is indeed an interesting question whether the training intensity may affect the quality of life in elderly patients undergoing CR, nevertheless, we feel that this would be beyond the scope of the present study.

Please see figure below for the relation between training intensity and quality of life:

What are the reasons that Copenhagen and Zwolle chose for high intensity interval trainings?

Co-authors from Copenhagen and Zwolle have themselves been involved in studies assessing benefits of high-intensity interval training (HIIT) and have implemented this training modality into clinical practice many years ago. The same applies to Bern, however, there, HIIT is offered to patients who prefer this training modality, which is not the case for many elderly patients. In our studies on HIIT we have not found HIIT to be superior to continuous endurance training for neither cardiac function (measured by echocardiography), peak VO2, chronotropic competence, nor heart rate variability (in press). In our view, many studies that have found HIIT to be superior to moderate intensity continuous training (MICE) with regard to exercise capacity outcomes have set MICE at intensity levels lower than what patients can easily achieve, which results in a somewhat “unfair” comparison, when you push one group but slow down the other.

**Reviewer #2:** This is an interesting paper with findings that could be clinically highly relevant. However, the reviewer did notice some issues in the methodology and results that require further (statistical) analysis, or at least significant reconsideration, to come to valid conclusions. Therefore, to increase the likelihood for publication, a major revision may be needed.

Introduction
It could be explained in greater detail to what extend VO2peak matters in older patients with CVD (e.g. prognosis, physical functioning, quality of life)?

Since we have published extensively on the importance of peak VO2 in our elderly cardiac cohort of the EU-CaRE study, we have chosen not to expand on this in the present study but rather to focus on how training intensity is related to peak VO2.

(please see following EU-CaRE publications(:

- Marcin T, Eser P, Prescott E, Mikkelsen N, Prins LF, Kolkman EK, Lado-Baleato Ó, Cardaso-Suaréz C, Bruins W, van der Velde AE, Peña Gil C, Iliou MC, Ardissino D, Zeymer U, Meindersma EP, Van't Hof AW, de Kluiver EP, Wilhelm M. Predictors of pre-rehabilitation exercise capacity in elderly European cardiac patients - The EU-CaRE study. Eur J Prev Cardiol. 2020 Nov;27(16):1702-1712;
- Prescott E, Eser P, Mikkelsen N, Holdgaard A, Marcin T, Wilhelm M, Gil CP, González-Juanatey JR, Moatemri F, Iliou MC, Schneider S, Schromm E, Zeymer U, Meindersma EP, Crocamo A, Ardissino D, Kolkman EK, Prins LF, van der Velde AE, Van't Hof AW, de Kluiver EP. Cardiac rehabilitation of elderly patients in eight rehabilitation units in western Europe: Outcome data from the EU-CaRE multi-centre observational study. Eur J Prev Cardiol. 2020 Nov;27(16):1716-1729.)

However, upon the reviewer’s suggestion we have added two references on the association of peak VO2 with mortality in the first sentence of the Introduction sentence (after “prognosis”).

5.	Hung RK, Al-Mallah MH, McEvoy JW, Whelton SP, Blumenthal RS, Nasir K, et al. Prognostic value of exercise capacity in patients with coronary artery disease: the FIT (Henry Ford ExercIse Testing) project. Mayo Clinic proceedings. 2014;89(12):1644-54.

6.	Vanhees L, Fagard R, Thijs L, Staessen J, Amery A. Prognostic significance of peak exercise capacity in patients with coronary artery disease. Journal of the American College of Cardiology. 1994;23(2):358-63.

Please include a study hypothesis?

Please note that the present study is a subanalysis of the observational EU-CaRE study whose primary aim was to compare the centres with regard to change in peak VO2 (*Prescott et al. 2020, Cardiac rehabilitation of elderly patients in eight rehabilitation units in Western Europe: Outcome data from the EU-CaRE multi-centre observational study. Eur J Prev Cardiol. 2020 Nov;27(16):1716-1729.doi:10.1177/2047487320903869. Epub 2020 Feb 26. PMID: 32102550*).

Hence, the present study should be considered as explorative and not as confirmatory. We believe that the aims are appropriate for this purpose.

Methods
What is ‘old’? For now, the cut-off of 65 years was used (which is arbitrary, but I understand the authors would select this age threshold), but in CR practice, 65 years is often the average age of the population (see for example reference 3). So I wonder whether similar results will be found if the authors would specifically focus on patients >75 years?

Thank you for pointing this out. Indeed, the cut-off 65 is somewhat arbitrary albeit often used. We included age as covariate within the model (S1 Table. Based on your comment, we added the interaction age (binary variable, <75 versus ≥75) * intensity (binary variable, above versus below VT) into the model which was not significant (p = 0.603). Therefore, we do not have any evidence that our results differ in patients aged above 75 compared to those below 75.

‘In each patient, we aimed to record HR during three trainings…’ should be ‘In each patient, we aimed to record HR during three training sessions…’?

Yes, thank you. We have changed this sentence accordingly (p.5).

Were changes in beta-blocker therapy allowed during CR? This could affect the training HR and consequently the statistical outcomes.

Yes, there were few changes in beta-blocker therapy between visit 1 and visit 2. Please see cross table below (0 = no beta-blocker therapy, 1 = beta-blocker therapy). Sixty-six patients had no beta-blocker at visit 1 but did have beta-blocker at visit 2, while 117 had beta-blocker at visit 1 but no beta-blocker at visit 2. The majority, however, namely 1153 patients, had beta-blocker at both visits. Unfortunately, we do not have information about the dose of treatment.

**vis2**

**vis1**	0	1

0	228	66

1	117	1153

A subanalysis without patients who changed beta-blocker treatment resulted in almost the same estimates as the full model. Please see model output of the subanalysis below and S1 Table (full model) for com-parison.

Model without patients who changed beta-blocker treatment from vis1 to vis2.

------------------------------------------------------------

Intercept	2.11 [ 1.24; 2.99] *

Training above VT1	0.56 [ 0.15; 0.96] *

Total training volume per CR [h]	0.07 [ 0.01; 0.12] *

Duration of cardiac rehabilitation [days]	0.00 [-0.00; 0.01]

baseline VO2 [ml/kg/min]	-0.08 [-0.13; -0.04] *

Age [years]	-0.07 [-0.10; -0.03] *

stable CAD (ref. CABG)	-1.34 [-2.20; -0.48] *

PCI (ref. CABG)	-1.51 [-1.99; -1.03] *

percutaneous HVR (ref. CABG)	-2.11 [-3.87; -0.35] *

surgical HVR (ref. CABG)	0.07 [-0.76; 0.90]

sex	0.55 [ 0.01; 1.08] *

Betablocker treatment	0.07 [-0.47; 0.62]

--------------------------------------------------------------------

AIC	3374.45

BIC	3438.28

Log Likelihood	-1673.22

Num. obs. 706

Num. groups: siteid	7

Var: siteid (Intercept)	0.14

Var: Residual	6.45

=============================================================

* 0 outside the confidence interval

S1 Table. Linear mixed model on change in peak VO2 [ml/kg/min] with

centre as random factor:

---------------------------------------------------------------------

Intercept	2.06 [ 1.28; 2.84] *

Training above VT1	0.62 [ 0.24; 1.00] *

Total training volume per CR [h]	0.06 [ 0.01; 0.12] *

Duration of cardiac rehabilitation [days]	0.00 [-0.00; 0.01]

baseline VO2 [ml/kg/min]	-0.09 [-0.13; -0.05] *

Age [years]	-0.08 [-0.11; -0.04] *

stable CAD (ref. CABG)	-1.31 [-2.10; -0.52] *

PCI (ref. CABG)	-1.50 [-1.95; -1.05] *

percutaneous HVR (ref. CABG)	-2.02 [-3.67; -0.37] *

surgical HVR (ref. CABG)	-0.13 [-0.90; 0.64]

sex	0.60 [ 0.10; 1.10] *

Betablocker treatment	0.08 [-0.38; 0.55]

---------------------------------------------------------------------

AIC	3852.50

BIC	3918.22

Log Likelihood	-1912.25

Num. obs. 808

Num. groups: siteid	7

Var: siteid (Intercept)	0.14

Var: Residual	6.42

=====================================================================

‘Alpha level was set at 0.05 for all analysis’: was this two-tailed?

Yes, thank you. The last sentence of the Statistical Analysis section in the Methods now reads: “Alpha level was set at 0.05 for all analyses (two-tailed for Wilcoxon-rank sum test).”

Were the patients allowed to execute strength training (older patients are often in need of strength training...)? If so, was this corrected for during the statistical analysis?

Four of our 8 centres offered also resistance training (Bern, Copenhagen, Ludwigshafen, Parma). An overview of the offered program characteristics has been published in Prescott E, Meindersma EP, van der Velde AE, Gonzalez-Juanatey JR, Iliou MC, Ardissino D, Zoccai GB, Zeymer U, Prins LF, Van't Hof AW, Wilhelm M, de Kluiver EP. A EUropean study on effectiveness and sustainability of current Cardiac Rehabilitation programmes in the Elderly: Design of the EU-CaRE randomised controlled trial. Eur J Prev Cardiol. 2016 Oct;23(2 suppl):27-40. (Table 1)

However, we did not adjust for resistance training because 1) we would not expect a major impact of resistance training on peak VO2 and 2) we did not record the heart rate / intensity of these resistance training sessions.

Results
Please mention n/centre in Table 2.

n/centre is reported in brackets in the first rows “frequencies” (e.g 165 of 203). This means that total number of patients in Bern were 203 but training heart rate measurement was available only in 165 patients.

In Table 2, total training volume should be expressed as peak effort training hours as well (sessions * duration/session * intensity). For now, the actual volume of exercise (expressed as hours) cannot be compared between the centres if the intensity also varied (to be determined by univariate correlations). In addition, would these peak effort training hours correlate with changes in VO2peak and thus affect the outcome from the regression analysis?

In addition to training intensity and training volume we have now included also “training dose” (total training volume [h] * training intensity [% of peak HR]) in Table 2 for better comparison.

For now, the authors compared patients training below or above VT1. I’m not surprised that patients exercising above VT1 improve better when compared with patients exercising below VT1 (below VT1 is really low…). However, between VT1 and VT2 a large HR range can be prevalent in some patients. So the question arises whether the effects of exercise intensity would be seen if patients would be categorized according to a different % of VT2 (under the assumption that the patients did not exercise above VT2)?
This is a very interesting question. However, we have not included further analyses with respect to the VT2 as we had even more missing values of VT2 than VT1. Also, VT2 may be missing especially in fragile patients who did not reach the VT2, so inclusion of % of VT2 would increase even more selection bias.

Nevertheless, based on your question we have performed the regression model with training HR in percent of the HR at VT2. The effect of the intensity based on the VT2 on change in peak VO2 was 0.02 [-0.00; 0.04], p = 0.07. In our opinion, the conclusion would be the same, namely that the training intensity in elderly may be overrated.

Discussion
Habitual physical activity was not assessed during CR: this could have affected the change in VO2peak and should be acknowledged as a limitation.

Yes, the reviewer is absolutely right, unfortunately, we did not assess habitual activity between visit 1 and visit 2. We have therefore added the following sentence to the limitations on p. 15:

*“Also, we did not assessed habitual physical activity outside of the CR program.”*

The authors mention: ‘According to the joint position statement of the European Association for Cardiovascular Prevention and Rehabilitation, the American Association of Cardiovascular and Pulmonary Rehabilitation and the Canadian Association of Cardiac Rehabilitation, the VT1 is reached at around 50-60% of peak VO2 and 60-70% of peak HR whereas the VT2 is reached at around 70-80% of peak VO2 and 80-90% of peak HR. [2] On average, the thresholds identified in this
study are slightly higher than these ranges, probably due to a lesser degree of exertion reached in these elderly patients.’ This actually is not an issue that should be considered as a study limitation: reference 3 reports similar thresholds…so they actually agree with literature. The reviewer believes rather the guidelines should change according to observations made in many CR centres.

This paragraph is not in the limitation section, rather it is intended to explain a potential mechanism leading to the discrepancies between our results and the ranges from the recently published Guidelines. Maybe future updates of the Guidelines will need to consider our and the findings of author studies.

**Reviewer #3**: This paper presents results from a prospective, multi-centre study investigating the cost effectiveness and sustainability of, and engagement in, cardiac rehabilitation (CR) programmes across several European countries. The analyses presented within this paper are a secondary analysis of the data, examining the intensity and volume of exercise completed during CR compared to current guidelines, and examine their association with improvements in directly measured V̇O2peak. A major strength of this study is the comparatively large sample size allowing for comprehensive multivariate modelling. The paper is novel in that it surveys exercise training variables within current CR programmes compared to international guidelines. Moreover, this paper is the first to examine the role of intensity and volume on cardiorespiratory fitness outcomes in a large cohort of older adults.
The paper is generally well written, though may benefit from minor edits as noted below.
General:
• Some inconsistencies in formatting of VO2 throughout.

Thank you for spotting the inconsistencies. We have adapted them accordingly.

• Incorrect formatting for abbreviations of physiological measures: VE requires subscript E, and VO2, VCO2 and VE all require an over-dot above the V to indicate values are per unit time (e.g. V̇O2 and V̇E).

We have changed these according to the reviewers suggestion.

• I strongly recommend referring to workload as work rate (abbreviated to WR in the results) as opposed to Watt – it is not common practice to refer to a variable by its units (e.g. the authors do not refer to heart rate as bpm)

Thank you for pointing this out, we have changed the abbreviation accordingly.

Introduction:
• Formatting of list for aims should present numbers as (1) and (2).

We have introduced numbering for the aims in the Introduction.

Methods:
• The authors should elaborate on, or provide citation for, contraindications to cardiac rehabilitation as part of their exclusion criteria

Contraindication to CR were set according to the position paper from the Cardiac Rehabilitation Section of the European Association of Cardiovascular Prevention and Rehabilitation. We have included following citation:

European Association of Cardiovascular Prevention and Rehabilitation Committee for Science Guidelines; EACPR, Corrà U, Piepoli MF, Carré F, Heuschmann P, Hoffmann U, Verschuren M, Halcox J; Document Reviewers, Giannuzzi P, Saner H, Wood D, Piepoli MF, Corrà U, Benzer W, Bjarnason-Wehrens B, Dendale P, Gaita D, McGee H, Mendes M, Niebauer J, Zwisler AD, Schmid JP. Secondary prevention through cardiac rehabilitation: physical activity counselling and exercise training: key components of the position paper from the Cardiac Rehabilitation Section of the European Association of Cardiovascular Prevention and Rehabilitation. Eur Heart J. 2010 Aug;31(16):1967-74. doi: 10.1093/eurheartj/ehq236. Epub 2010 Jul 19. PMID: 20643803.

• The methods state that thresholds were only analysed by a single author. The authors should provide a rationale for this and note the experience/expertise of the lead author to reliably identify ventilatory thresholds. Moreover, did the authors conduct any assessment of reliability for a random subset of CPET outputs? If not, I believe this should be undertaken and the agreement between authors presented within the results. As this is a major outcome for this study it is imperative the authors demonstrate the accuracy and reliability of their analyses.

The clinicians of the respective centres determined the ventilatory thresholds as part of clinical routine. However, we felt that this would increase the risk of a systematic bias, e.g., some centers would achieve systematically higher thresholds than others. In order to apply the same criteria to all patients from all centres we chose one single investigator to set all thresholds in a random and blinded fashion (on randomly ordered blinded images of filtered and smoothed raw data). We believe that this should be viewed as a strength rather than a limitation. However, we agree with the reviewer that some quality check was necessary and have added a sub-analysis of 200 CPETs analysed by another experienced cardiologist (MW) to assess inter-rater reliability (Supplement Figure 1). With an Intraclass-Correlation-Coefficient of 0.92 for VT1 and 0.97 for VT2 the reliability was excellent.

• 30 s moving average for V̇O2, HR and Watts at VT1 and VT2 requires further clarification – was this taken as the 30 s prior or 30 s following, or 15 s either side?

It was a centered moving average. It reads now:

*“A centered moving average over 30 s for V̇O2, HR and WR was recorded at VT1, VT2 and peak exercise.”*

• When determining whether a patient’s mean exercise intensity was above or below VT1, how was specifically determined? Did the authors calculate the mean HR for each session then compare the mean of these means to the HR at VT1? This requires further clarification.

Yes, the patient means of the mean training heart rate was compared to the HR at VT1. It reads now on p.6:

*“The mean training HRs from a patient’s monitored training sessions were averaged to one single mean training HR.”*

Results:
• The authors should undertake secondary confirmation of thresholds identified using a second author. Given the large sample size this may be completed as a random subsample of 15-20% of participants. The authors should then report the level of agreement in identifying VT1 and VT2 in this subsample.

*As already described above, based on your and reviewer 1’s suggestion we have added an inter-rater reliability analysis of 200 CPETs by an experienced cardiologist (MW) to the supplements. Level of agreement was excellent.*

• Figure 1 is difficult to interpret. This could be made clearer by additional descriptions for the three lowest boxes describing what the n values represent (i.e. No. of pts with both VT1 and VT2, n = 768) [I am assuming that is what this box represents?]

Thank you for your feedback. We have adapted Figure 1, namely we have changed some headers in the boxes and included superscripts to explain some of the boxes in the figure legend:

***«Fig 1: Flow-chart of available cases****: N represent number of patients having data to training intensity and/or ventilatory thresholds and/or change in peak* V̇O_2_*.
^a^ Number of patients with available training intensity and identified VT_1_; ^b^Number of patients with available training intensity and identified VT_2_; ^c^ Number of patients with available training intensity and identified VT_1_ and VT_2_.»*

• I believe a total cohort version of Figure 2 would be beneficial to include in the results. This could be included as an additional figure rather then adding further complexity to Figure 2.

Thank you for your suggestion. However, we believe that an additional Figure to the total cohort would overload the manuscript and as Figure 2 shows the results by centres with similar patient numbers, it can extrapolated to the total cohort.

• Legibility of Figure 2 could be improved by reducing line thickness of the boxplots and size of outlier symbols. Further, consider a different (lighter) colour scheme for the green and red boxes as these colours reduce the contrast with the box outline.

We have now uploaded the Figure as a tiff file, which has improved legibility (regarding line thickness and outlier symbols) than the previous vector graphic pdf file.

• There are abbreviation inconsistencies in caption for Figure 2.

Thank you for pointing these out. We have changed them accordingly.

• Can the authors include an additional column in Table 2 for the total cohort. This should be placed before the column for Bern. Naturally, present data for frequencies and median/IQR sections only.

We have included a column for the total cohort as suggested. Additionally, we have corrected the values for the upper quartile.

• In Table 2, footnote markers for a and b should be swapped – currently b comes before a.

Thank you, we have changed them accordingly.

Discussion:
• Final statement in ‘Training intensity’ subsection could be elaborated on. The authors should clarify if they suggest that intensity is not an important consideration for exercise prescription in this population – if not, what should clinicians focus on?

Please see answer for following comment.

• The self-paced exercise intensity subsection should be expanded to include discussion regarding perceptually-regulated exercise (i.e. RPE regulated). In practice, there is a continuum of self-regulated/self-paced exercise intensity. The authors have discussed affect-regulated exercise which encompasses exercising at an intensity that ‘feels good’ or elicits a positive affective response. In most circumstances, this is method of regulating exercise intensity introduces a high level of subjectivity; the clinician cannot effectively guide the patient to exercise at a low, moderate or high intensity. In contrast, with RPE-regulated exercise provides scope for patient subjectivity but still allows the clinician to direct the patient to different general intensities. This should be discussed in reference to the results of the present study that suggest that the intensity may not be important and that exercise at intensities that elicit a positive affective response may (only some emerging data is available) be associated with better attendance and compliance in CR programmes.

Thank you for your input. We have included the following paragraph on p.16:

*“Already widely established in clinical routine is the exercise intensity prescription according to self-rated perceived exertion using the BORG scale (2). This method, while providing scope for patient autonomy, allows clinicians to direct patients towards different intensity ranges.“*

• The limitations section should not that thresholds used in analyses were identified by only 1 author. The authors should consider and discuss the impact of erroneous threshold values on their outcomes.

Since we have now added the inter-rater reliability analysis with excellent agreement we feel that this is no longer a limitation. As previously described, we consider it as a strength that one investigator identified all ventilatory thresholds of all patients in a blinded fashion. Of course, variation in the setting of ventilatory thresholds may affect the results, however, due to the blinding of the investigator with regard to patient, time point and centre introduction of systematic bias can be excluded. In our opinion, a more extensive discussion about the ventilatory threshold setting would be beyond the scope of the manuscript.
